# Mid-esophagus unresectable cancer treated with a low cost stent. First experience

**DOI:** 10.1186/1756-0500-4-486

**Published:** 2011-11-10

**Authors:** Valter N Felix, Alaor Caetano, Jose P Cipullo, Emiliano C Almodova, Wagner Colaiacovo, Aldenir F Zamboti

**Affiliations:** 1 Department of Gastroenterology, Surgical Division, University of São Paulo, School of Medicine (FMUSP). Rua Frei Caneca, 1407, c. 221. São Paulo, ZC 01307-909, Brazil; 2 Multidisciplinary and Endoscopy Service, Rio Preto School of Medicine (FAMERP). Avenida Brigadeiro Faria Lima, 5416-São José do Rio Preto, ZC 15090-000, Brazil; 3 Department of Endoscopy, Cancer Hospital. Rua Antenor Duarte Vilella, 1331-Barretos, ZC 14784-400, Brazil

## Abstract

**Background:**

In the cancer of the esophagus, with recent technologic advances, self-expanding metal stents (SEMS) are at the forefront of the armamentarium for re-establishing luminal patency. Weighed against the numerous advantages of stents are the import conditions and the cost. In light of this, we tested new low cost prostheses having the basic needs and characteristics to aim a significant benefit to poor people having advanced esophageal cancer, in a Brazilian regional public hospital.

**Methods:**

This initial experience included fifteen patients (eleven men and four women, 55 ± 6.17 years old), presenting esophageal cancer, located at the medium third of the thoracic esophagus, extending for 5.5-8 cm long, not suitable for surgical procedure because they had been staged on fourth grade of the disease, two of them having fistula communicating esophagus to respiratory tree. The stents were placed under endoscopic and fluoroscopic guidance, after attempting an esophageal dilatation. An appropriate covered stent was then deployed, twelve of 10 cm and three of 13 cm in length. A chest X-ray was done 2 h after the procedure and a barium swallow was performed within 12 hours. Seven days and monthly until complete a six month follow-up after the procedure the patients were questioned about presence of pain, regurgitation, heartburn, cough, and their alimentary behavior.

**Results:**

There were no severe complications and transient mild chest pain resolved until the seventh day after the stent deployment. Chest X-ray demonstrated expansion of the stent in all patients. In 2 cases of fistula, a barium swallow showed its complete sealing. The completion of the proposed follow-up was not achieved in three cases, limited by the patient's death until the third month, due to cancer progression. Recurrent dysphagia to paste food accounted for by tumor overgrowth proximal or distal to the stent and stent migration were not observed in the series.

**Conclusions:**

The new low cost endoprostheses is effective and forthcoming increased experience and prospective trials including questionnaires to analyze quality of life will allow for more informed decisions tailoring to a particular patient situation or to unexpected complications.

## Background

In the cancer of the esophagus, for both palliation of obstructive symptoms and continued oral intake, the placement of an endoprostheses can greatly enhance the quality of life of the patient. With recent technologic advances, self-expanding metal stents (SEMS) are at the forefront of the armamentarium for reestablishing luminal patency.

Progressive dysphagia, initially to solids, later to liquids and secretions, is one of the most frequent debilitating complaints. The prognosis is dismal: more than 60% of patients are not operable at the time of diagnosis, and the overall 5-year survival is lesser than 5% [1]. Palliation is the only realistic therapeutic option for these patients. Available palliative treatment modalities include chemotherapy, radiation therapy, esophageal bypass, esophageal dilation, multipolar electrocoagulation, laser treatment, injection of sclerosing agents, photodynamic therapy, and esophageal endoprostheses.

Esophageal endoprostheses have been increasingly used in this setting and currently comprise semi rigid plastic stents and self-expanding metal mesh stents. Both types of endoprostheses provide rapid and lasting relief of dysphagia.

However, plastic stents require general anesthesia for placement and carry a high complication rate, with stent-related mortality ranging from 4% to 13% [2]. With the ever decreasing diameters of delivery systems, self-expanding metal mesh stents can be easily inserted in an outpatient procedure using conscious sedation. And, with advancement in user-friendly designs, there has been continued improvement in the safety and ease with which these devices can be inserted.

In past decade, several prospective randomized trials demonstrated a higher effectiveness and much lower complication (0% vs. 21%) and mortality (0% vs. 15.8%) rate for SEMS vs. plastic stents, respectively [3,4]. SEMS were also shown to be more cost-effective, considering the shorter hospital stay and lower mortality [2]. Additional applications for SEMS, such as treatment of bronchoesophageal fistulas, have emerged.

Earlier, uncovered (bare metal) designs allowed ingrowths of tumor through wire mesh, causing the stents to anchor in place. In contrast, covered versions effectively prevent such tumor ingrowths but have a higher rate of migration. Because the goal of SEMS is to maintain luminal patency, there must be a favorable balance between migration rates and prevention of tumor ingrowths. Current designs generally incorporate a covered midsection with uncovered flared ends that provide an anchoring mechanism.

Data comparing the effectiveness, complication rates, and costs for different stent types are limited. Currently, the choice of particular SEMS depends primarily on the physician's experience and preference. Stent technology and design continue to evolve with the goal of improving safety parameters [5].

In order to comprehensively assess the relative merits of the different palliative treatments of malignant dysphagia, health economic aspects have to be incorporated. Weighed against the numerous advantages of stents, particularly in the Brazilian public health net, are the import conditions and the cost. In light of this, we sought to employ immediately, as soon as approved by ANVISA (Brazilian Official Office that certifies health products) a prostheses (registration number 10159030069) that would enable, in the case of all those providing services to patients with unresectable esophageal cancer, prompt and accessible action in the treatment of these severely ill patients, at a cost of less than US$ 1,500, quite reasonable when compared with the average cost of £ 4,900 in the palliative care of patients with inoperable esophageal cancer by other methods [5], contributing objectively to Brazilian public health.

This prospective study was proposed to introduce the use of the new stent in a regional public hospital and to verify its first clinical results.

## Methods

Fifteen patients (eleven men and four women, 55 ± 6.17 years old), presenting esophageal cancer, located at the medium third of the thoracic esophagus, extending for 5.5-8 cm long were admitted to the Cancer Hospital (Barretos-São Paulo). The Ethical-Scientific Commission of the Hospital approved the study.

They were not suitable for surgical procedure because they had been staged on fourth grade of the disease, two of them having fistula communicating esophagus to respiratory tree. The complete demographic, clinical, imaging (barium study, chest and abdomen's contrast-enhanced computed tomography), endoscopic and histological findings (squamous cancer) had been previously recorded and the inclusion criteria was locally advanced unresectable cancer, with the patients having ability to swallow liquids only. Their body mass index average achieved 18.16 ± 2.17 Kg/m^2 ^and serum albumin, 2.9 ± 0.51 g/100 mL.

The self-expandable stent for the esophagus is made of braided SE508 nitinol, a binary alloy suitable for superelastic monofilament wires, composed of Ni (55.8%), Ti (44.17%), O (0.02%), H (0.0001%) and C (0.0039%), forming diamond shapes. It has nitinol wires of 0.125 mm, 0.150 mm, 0.210 mm in diameter, respectively, in grafts of 7, 10 and 13 cm in length.

**Figure 1:**
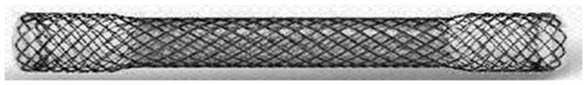
**The new self-expandable stent for the esophagus**.

The stent has an hourglass shape. Thus, its diameter increases gradually, from the 20 mm of its tubular body, up to 22 mm, along the 15 mm length of the outer parts of its tapered ends finished with gold markers. The polyurethane coating (ASTM D-2240) has a hardness of 75 (Shore), tensile strength of 7500 psi (ASTM D-412) and 500% elongation (ASTM D-412). The stent is coated but retains its conical ends of exposed wire (Figure 1).

The self-expanding stent is inserted into a catheter release mechanism and coaxial type sheath which has as its components: handle, metal tubing, metal tubular lock, hemostatic valve, sheath cover, an extractor to release the prosthesis and a radiopaque silicon atraumatic tip. The release mechanism, with an internal diameter allowing passage of a guide wire of up to 0.035 inch, is composed of two coaxial bodies, the insides of which serves as internal housing for the stent and guide wire passage. It is equipped with radiopaque markers on its distal part for guidance on release and its proximal part is embedded in the stem of stainless steel cable.

After obtaining informed consent, all patients were submitted to the procedure and hospitalized for 24 h of observation, to provide a safer care after employ new kind of stent. The procedure was performed under local anesthetic spray, using propofol for intravenous sedation. The stents were placed under endoscopic and fluoroscopic guidance. First, the guide wire was introduced and an esophageal dilation was attempted (12 mm) under an image intensifier and then the location and length of the stenosis was defined endoscopically. The stricture was marked by contrast injection in its extremities. An appropriate covered stent was then deployed, twelve of 10 cm and three of 13 cm in length.

A chest X-ray was done 2 h after the procedure to check the expansion of the stent and to look for any evidence of surgical emphysema or pneumothorax. A barium swallow was performed within 12 hours.

Oral liquids were allowed after the chest X-ray and the barium swallow, and pastes were authorized at the outcome. All patients were advised to keep the head end elevated during sleeping to prevent aspiration, to chew food properly and to avoid consistent solid food. Adjuvant therapy was not recommended due to the poor clinical conditions of the patients.

Seven days and monthly until complete a six month follow-up after the procedure the patients were questioned about presence of pain, regurgitation, heartburn, cough, and their alimentary behavior.

## Results

The mean ± SD propofol doses (mg/min per kg weight) used was 0.14 ± 0.02. Stent placements were not associated with any immediate or late technical complications. There were no severe complications, such as fistula formation, sepsis, or death, due to esophageal stent placement and transient mild chest pain, reported by all, was controlled with paracethamol (750 mg twice a day) resolved until the seventh day after the stent deployment.

**Figure 2:**
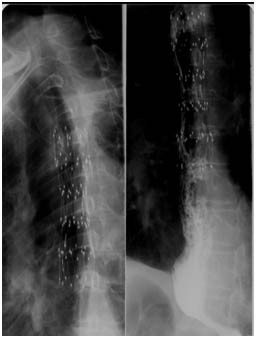
**Chest X-ray showing expansion of the stent, 2 h after the procedure (left) and a barium swallow performed 12 h after the procedure, documenting complete sealing of a fistula (right)**.

Chest X-ray demonstrated expansion of the stent in all patients and absence of esophageal perforation signs. In 2 cases of fistula, a barium swallow showed its complete sealing (Figure 2). No severe chest pain, bleeding or respiratory distress was reported. Regurgitation and heartburn didn't occur and cough diminished significantly seven days after the sealing of the fistulas, enhancing the quality of life of these terminal patients.

The completion of the proposed follow-up was not achieved in three cases (stents of 13 cm in length; two with esophageal fistula to respiratory tree), limited by the patient's death at 4-12 weeks after the procedure, due to cancer progression, but until death they had preserved a free alimentary route without food impaction or aspiration. Recurrent dysphagia to paste food accounted for by tumor overgrowth proximal or distal to the stent and stent migration were not observed in the series until the sixth month.

## Discussion

Palliation of malignant dysphagia is the mainstay of therapy in patients with incurable esophageal cancer. SEMS placement is a well-established application in this group of patients, mainly when cancer is located in the thoracic esophagus, away from the cardia and the upper esophageal sphincter (UES). Stents located through the cardia have a high migration index and often cause symptomatic reflux and those until 2 cm near the UES determine continuous discomfort.

After undergoing esophageal stent placement, patients need to modify their diet to prevent large boluses of food from becoming impacted within the stent causing immediate or late aspiration of the residues collected above. We didn't have such complications, but the patients were all instructed to sleep with the head of the bed raised and to avoid consistent solid food, as a prophylactic care.

Esophageal stent placement includes endoscopic assessment, guide wire insertion, tumor dilation, and stent deployment. Esophageal dilation is generally done prior to stent insertion; precise requirements for dilation depend on stent type.

Prior to stent placement, a complete endoscopic examination should be performed to assess the proximal and distal extent of the stricture. Dilation may be necessary as a first step to allow a complete exam, but the optimal degree of dilation prior to SEMS placement has not been established. With the current small delivery systems, it is generally recommended to dilate to at least 12 mm if possible, as we have done; under fluoroscopic guidance, the most useful and safest method to perform a stent placement [1,2], a guide wire can be safely passed preceding dilation. The location of the tumor, at the medium third of the thoracic esophagus, in all the patients, and all those care measures mentioned above certainly guaranteed the null rate of severe complications of this series, but greater casuistic maybe will provoke inevitable unsuccessful cases.

When stenting mid-esophageal malignant strictures, as in this series, it is wise to leave the final dilator in place for 30-60 seconds to assess for respiratory compromise due to tumor displacement. If such compromise occurs, tracheal stenting to ensure airway patency should precede esophageal stent placement, what was not necessary in any of our cases. A chest X-ray was performed after stent placement to assess stent location and exclude complications. When the stent is properly positioned, the esophageal stricture induces a "waist" at the center of the stent immediately after deployment.

Procedure-related complications after stent placement, which weren't observed in this initial series, occur in 5% to 10% of patients and mainly consist of perforation, aspiration pneumonia, fever, hemorrhage, and severe pain. Minor complications, which are reported by 10% to 20% of patients, include mild retrosternal pain, as our patients reported, and GE reflux symptoms. Delayed complications and recurrent dysphagia to paste food following stent placement are important problems and have been reported to occur in 30% to 40% of patients. Delayed complications include hemorrhage, fistula formation, stent migration, tissue ingrowths or overgrowth, and food-bolus obstruction [6]. Possibly a great part of these problems could be avoided reserving the stent to mid esophagus tumors, not completely obstructive, as in this series, avoiding endoprostheses and performing only a gastrostomy in extremely severe grade of the disease.

The decreasing diameters of delivery systems make perforation a rare occurrence, generally related to pre-stent dilation. Chest pain seems to be related to the expansible force of the stent, and generally resolves within some days, as we observed. Most patients with chest pain may be managed adequately with routine analgesics, as we did.

A retrospective study compared two different types of SEMS (uncovered and partially covered) for palliative treatment of 152 patients (uncovered 54 and partially covered 98) with inoperable malignant stenosis of the esophagus and cardia [7]. Overall, 88% of patients with partially covered stents and 54% with uncovered stents were free of symptoms during follow-up (*P *< 0.0001). Although the rates of stent migration were lower in the uncovered stents group (0 vs. 10%, *P *= 0.03), tumor or granulation tissue ingrowths (100 vs. 53%, *P *< 0.0001) and restenosis causing recurrent dysphagia (37 vs. 8%, *P *< 0.0001) were significantly higher in the uncovered stents group.

It has now convincingly been shown that fully or partially covered metal stents, our stent choice, give better long-term palliation of malignant dysphagia than uncovered stents. The technical success rate for placement of partially or fully covered metal stents is close to 100%. Almost all patients experience rapid improvement of dysphagia within a few days [8], as in our initial series.

The endoscopic placement of covered SEMS is the treatment of choice for malignant esophageal fistulas. The quality of the evidence for malignant fistula closure with SEMS is moderate and the strength of the recommendation is strong (given the paucity of alternatives). When placing stents for this indication, it is imperative to assess for airway compromise during dilation. If respiratory distress occurs, tracheal stenting should be performed prior to placement of the esophageal stent. Fortunately we didn't have this problem and the complete closure of the fistula was showed in the barium swallow performed 12 h after the procedure. As a consequence, the patients had a grateful relief of the cough.

In three cases, the duration of follow-up was limited to just 4-12 weeks by occurrence of the patient's death due to cancer progression. This fact correlates with the advanced disease stage that these patients had prior to esophageal stent placement (13 cm in lenght), two of them showing an esophageal fistula to respiratory tree. It is likely that esophageal stent placement would be even more beneficial if done earlier in the disease course.

Our first experience reveals that the majority of stents certainly could be placed on an outpatient basis. The patient should be observed for 2 hours after the procedure, and if clinically stable and well receiving liquids via oral route, could be discharged to home with dietary instructions.

## Conclusion

The new low cost endoprostheses is effective and forthcoming increased experience and prospective trials including questionnaires to analyze quality of life will allow for more informed decisions tailoring to a particular patient situation or to unexpected complications.

## List of Abbreviations

SEMS: self-expanding metal stents; ANVISA: Brazilian Official Office that certifies health products; ASTM: American Society for Testing in Materials; SD: Standard deviation; Ni: Nickel; Ti: Titanium; O: Oxygen; H: Hydrogen; C: Carbon; psi: Pounds/square inch.

## Competing interests

The authors declare that they have no competing interests.

## Authors' contributions

VNF designed the study and wrote the final manuscript. AC followed-up the patients. JPC participated in the design and in the coordination of the study. ECA and WC performed the stent placements. AFZ participated in the stent placement and in the follow-up of the patients. All authors read and approved the final manuscript.

## References

[B1] Sagar PM Gaupera T Sue-Ling H McMahon MJ Johnston D An audit of the treatment of cancer of the oesophagus Gut 1994 35 941 945 10.1136/gut.35.7.941 7794305 PMC1374841

[B2] Knyrim K Wagner HJ Bethge Klaus N Keymling M Vakil N A controlled study of an expansile metal stent for palliation of esophageal obstruction due to inoperable cancer N Engl J Med 1993 329 1302 1307 10.1056/NEJM199310283291803 7692297

[B3] De Palma GD Di Matteo E Romano G Fimmano A Rondinone G Catanzano C Plastic prosthesis versus expandable metal stents for palliation of inoperable esophageal thoracic carcinoma: a controlled prospective study Gastrointest Endosc 1996 43 478 482 10.1016/S0016-5107(96)70290-0 8726762

[B4] Roseveare CD Patel P Simmonds N Goggin PM Kimble J Shepherd HA Metal stents improve dysphagia, nutrition and survival in malignant esophageal stenosis: a randomized controlled trial comparing modified Gianturco Z-stents with plastic Atkinson tubes Eur J Gastroenterol Hepatol 1998 10 653 657 9744693

[B5] Shenfine J McNamee P Steen N Bond J Griffin SM A Randomized Controlled Clinical Trial of Palliative Therapies for Patients With Inoperable Esophageal Cancer Am J Gastroenterol 2009 104 1674 1685 10.1038/ajg.2009.155 19436289

[B6] Homs MY Siersema PD Stents in the GI tract Expert Rev Med Devices 2007 4 741 752 10.1586/17434440.4.5.741 17850208

[B7] Saranovic Dj Djuric-Stefanovic A Ivanovic A Masulovic D Pesko P Fluoroscopically guided insertion of self-expandable metal esophageal stents for palliative treatment of patients with malignant stenosis of esophagus and cardia: comparison of uncovered and covered stent types Dis Esophagus 2005 18 230 238 10.1111/j.1442-2050.2005.00484.x 16128779

[B8] Vakil N Morris AI Marcon N Segalin A Peracchia A Bethge N Zuccaro G Bosco JJ Jones WF A prospective, randomized, controlled trial of covered expandable metal stents in the palliation of malignant esophageal obstruction at the gastroesophageal junction Am J Gastroenterol 2001 96 1791 1796 10.1111/j.1572-0241.2001.03923.x 11419831

